# A novel lncRNA n384546 promotes thyroid papillary cancer progression and metastasis by acting as a competing endogenous RNA of miR-145-5p to regulate AKT3

**DOI:** 10.1038/s41419-019-1637-7

**Published:** 2019-06-03

**Authors:** Jiajia Feng, Qinyi Zhou, Hongliang Yi, Shiyin Ma, Dawei Li, Yanan Xu, Jiadong Wang, Shankai Yin

**Affiliations:** 1 Department of Otolaryngology Head and Neck Surgery, Shanghai Jiao Tong University Affiliated Sixth People’s Hospital, Yishan Road 600, Shanghai, 200233 China; 2 Shanghai Key Laboratory of Sleep Disordered Breathing, Yishan Road 600, Shanghai, 200233 China; 3 Otolaryngological Institute of Shanghai Jiao Tong University, Yishan Road 600, Shanghai, 200233 China; 4 Department of Head and Neck Surgery, Renji Hospital, School of Medicine, Shanghai Jiaotong University, Shandongzhong Road 145, Shanghai, 200001 China; 5 Department of Otolaryngology, the First Affiliated Hospital, Bengbu Medical College, Changhuai Road 287, Bengbu, 233004 Anhui China

**Keywords:** Thyroid cancer, Cell growth

## Abstract

Long noncoding RNAs (lncRNAs) are emerging as important regulators in the development of cancer cells. However, the role and mechanisms of most lncRNAs in papillary thyroid carcinoma (PTC) remain unknown. In this study, we investigated lncRNA expression profiles of PTC using RNA-seq in two groups of PTC tissues and adjacent normal tissues, and validated by real-time PCR analysis in another 53 pairs of tissues. We identified a novel lncRNA, n384546, which is highly expressed in PTC tissues and cell lines. n384546 expression was associated with clinicopathological features of PTC patients, such as tumor size, lymph node metastasis, and TNM stage. Functionally, knockdown of n384546 inhibited PTC cell proliferation, invasion, and migration both in vitro and in vivo. In addition, we identified miR-145-5p as a key miRNA target of n384546 using online bioinformatics tools. Anti-miR-145 could partially reverse the effects of n384546 knockdown. Furthermore, we found that n384546 could regulate the expression of AKT3 by sponging miR-145-5p, which was confirmed using an in vitro luciferase assay. In conclusion, we validated n384546 as a novel oncogenic lncRNA in PTC and determined that the n384546/miR-145-5p/AKT3 pathway contributes to PTC progression, which might be used as potential therapeutic targets for PTC patients.

## Introduction

Thyroid cancer is the most common endocrine malignancy, and its incidence worldwide has increased rapidly over past several decades. In China, incidence of thyroid cancer is 6.6 per 100,000 persons according to the Chinese Cancer Registry, and thyroid cancer has become the sixth most common malignant tumor in the female population of China^1^. The most common type of thyroid cancer is papillary thyroid carcinoma (PTC), which accounts for close to 85% of all thyroid cancer^2^. Most patients with PTC can be cured by traditional clinical managements such as thyroidectomy, radioiodine, or TSH suppression therapy. Although the 5-year overall survival rate of PTC patients is about 95%, tumors can metastasize into distant organs and lymph nodes, resulting in poor prognosis and high reoccurrence in some patients^3,4^. Thus, it is of great importance to investigate the underlying molecular mechanisms to improve the diagnosis and prognosis of PTC patients.

Long noncoding RNAs (lncRNAs) are generally delimited as transcripts longer than 200 nucleotides which have no protein-coding ability. Only ~2% of the human genome encodes proteins, whereas at least 75% is transcribed into noncoding RNAs, most of which are lncRNAs^5^. In recent years, lncRNAs are becoming known as key regulators of various physiological process, including chromatin remodeling, X chromosome imprinting, cell differentiation, and the pathogenesis of various human diseases^6–8^. Accumulating studies suggest that abnormally expressed lncRNAs may play vital roles in the development of various types of cancers, and recent studies suggest that certain lncRNAs could serve as biomarkers for tumor diagnosis and potential targets for treatment^9–12^. There are growing research findings showing that lncRNAs participating in the development of PTC, though roles of most lncRNAs in PTC remains unknown^13–16^.

In our research, high-throughput RNA sequencing (RNA-seq) was utilized to explore lncRNA expression profiles of PTC tissues and adjacent normal tissues. RNA-seq results were validated using quantitative real-time PCR (qRT-PCR). We found one novel lncRNA, n384546, which was the most significantly upregulated lncRNA in tumor tissues compared with normal tissues. Overexpression of n384546 was significantly related to clinicopathological features of PTC patients including tumor size, lymph node metastasis, and TNM stage. We selected n384546 as our lncRNA of interest and explored its effects and mechanisms on the progression of PTC. In vitro experiment showed silencing of n384546 inhibited PTC cell proliferation, migration and invasion, and promoted cell apoptosis. In vivo experiment confirmed tumor growth was suppressed after n384546 knockdown. In addition, n384546 was found to exhibit oncogenic properties by sponging miR-145-5p and then mediating the expression of AKT3. Taken together, the current study identified that lncRNA n384546 may be a novel biomarker for PTC diagnosis and treatment.

## Results

### lncRNA expression profile in PTC using RNA-seq

**Fig. 1:**
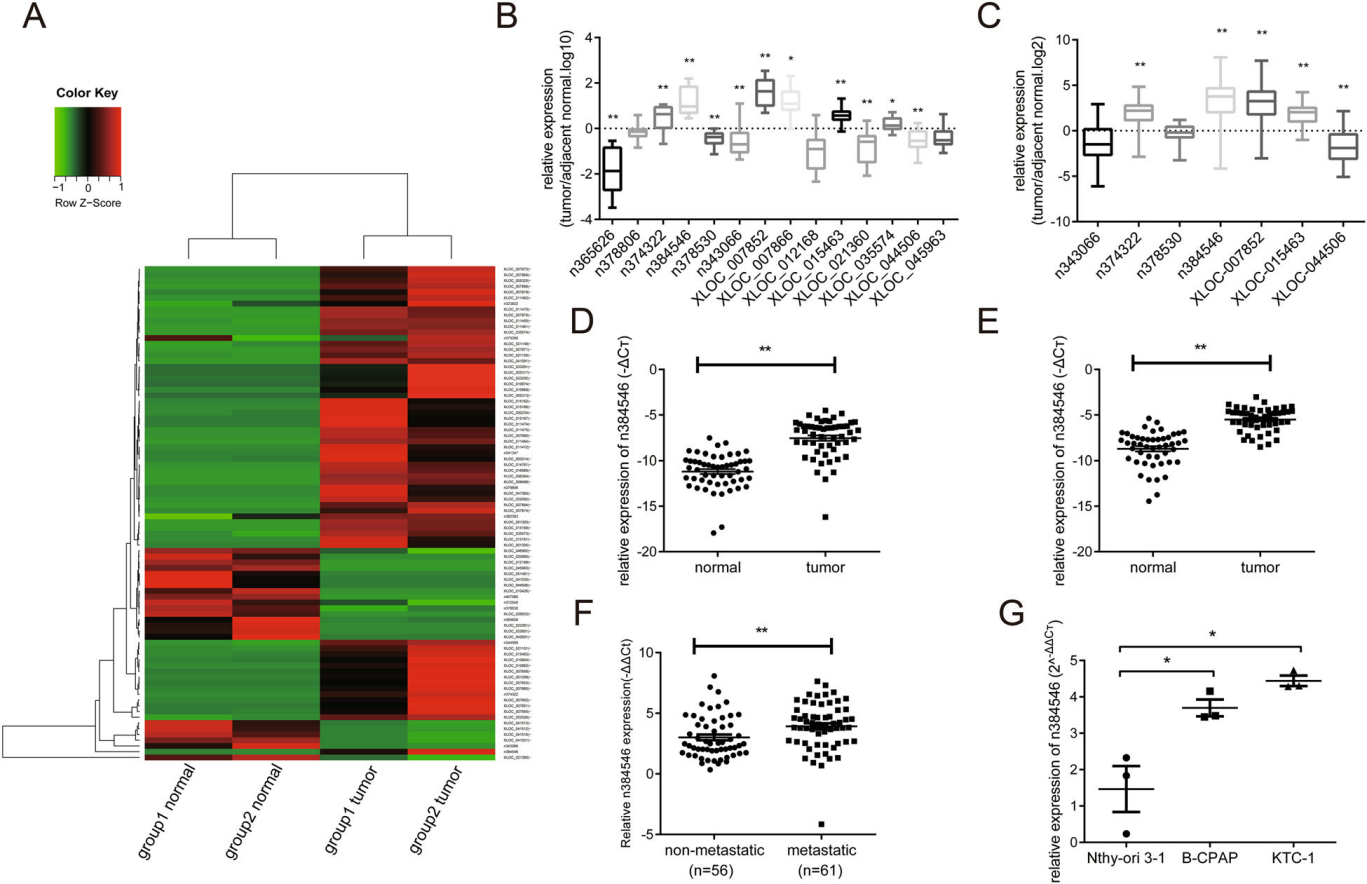
LncRNA n384546 is upregulated in PTC tissues and cells. **a** Hierarchical clustering analysis of 86 lncRNAs that were differentially expressed between PTC samples (tumor) and adjacent normal samples (normal) (>2.0-fold, *p* < 0.05). **b** Validation of the expression of 14 lncRNAs in 16 pair samples of PTC and adjacent normal tissues was determined by qRT-PCR. **c** The expression of the seven most differentially expressed lncRNAs in another 53 pairs of samples was determined by qRT-PCR. **d** LncRNA n384546 expression in 53 pair samples of PTC and adjacent normal tissues. **e** LncRNA n384546 expression in another cohort of 48 PTC patients. **f** Relative expression of lncRNA n384546 in PTC tissues without and with lymph node metastasis. **g** Relative levels of n384546 in normal thyroid cell Nthy-ori 3-1 and two types of PTC cells, B-CPAP and KTC-1, were determined by qRT-PCR. Error bars indicate the mean ± SEM. Data in (**e**) represent the mean ± SEM of three separate experiments. *p < 0.05, **p < 0.01 in paired Student’s *t* test (**b**–**f**) and independent Student’s *t* test (**g**)

Using RNA-seq, the predicted lncRNAs were classified and annotated. Filtered by fold change and *p* value (fold change >2 or fold change <0.5, *p* < 0.05), a total of 486 lncRNA transcripts (92 known lncRNAs and 395 new lncRNAs) were differentially expressed in tumor and normal samples in group 1, and a total of 568 lncRNA transcripts (95 known lncRNAs and 473 new lncRNAs) were differentially expressed in group 2. A total of 86 lncRNA transcripts were differentially expressed in both groups, and among the lncRNAs, 64 were upregulated and 22 were downregulated (Fig. 1a). The complete dataset was deposited into Gene Expression Omnibus (GSE124841).

To validate RNA-seq results, we selected seven upregulated lncRNAs and seven downregulated lncRNAs with high differential expression and analyzed their relative expression levels in the tissues of the same patients which were used for the RNA-seq study (16 pairs of PTC and corresponding normal thyroid tissues) using qRT-PCR. The expression of 11 lncRNAs were significantly different, which was consistent with the RNA-seq results. However, the expression levels of the other three lncRNAs in cancer tissues were not different from those in normal tissues, which did not match the RNA-seq results (Fig. 1b). In general, the expression trends of the validated lncRNAs are consistent with those of RNA-Seq.

### Novel lncRNA n384546 is upregulated in PTC

For further select the lncRNA which plays critical roles in the initiation and progression of PTC, the expression of seven lncRNAs which have significant difference in previous validation experience was analyzed in tissues of another 53 PTC patients using qRT-PCR. Results showed that n384546 was the most significantly dysregulated lncRNA in PTC tissues compared with adjacent normal thyroid tissue (Fig. 1c, d). The noncode v5.0 database shows that n384546 is a 3513-bp transcript with two exons and is localized on the reverse strand of chromosome 4: 172987196–172995762, and its expression in thyroid is the highest of all human tissues in the Human Body Map (Fig. S1). We further examined the expression of n384546 in another cohort of 48 patients, and the result was consistent with the previous result (Fig. 1e). Moreover, compared with patients without metastasis, n384546 in PTC tissues from patients with cervical lymph node metastasis was obviously upregulated (Fig. 1f). Then, we performed a correlation analysis between the expression level of n384546 and clinicopathological features of PTC patients to explore the possible role of n384546 in PTC. In Table 1, the median of n384546 expression was determined, and patients with expression below the median were placed in the low level group and patients with expression above the median were placed in the high level group. We found that high level of n384546 expression was correlated with a larger tumor size (>2 cm) (*p* = 0.003), positive lymph node metastasis (level VI) (*p* < 0.001), positive lymph node metastasis (level II, III, IV) (*p* = 0.005) and high TNM stage (*p* = 0.039). No significant relationship between n384546 and other clinical features such as age and gender was found. Then, n384546 expression level in two PTC cells (B-CPAP and KTC-1) and one human normal thyroid epithelial cell (Nthy-ori 3-1) was examined using qRT-PCR. As shown in Fig. 1g, we found that compared with Nthy-ori 3-1 cells, B-CPAP and KTC-1 cells have higher expression levels of n384546. In summary, these results suggested that upregulated n384546 might contribute to the carcinogenesis of PTC.

**Table 1:** Correlation between n384546 expression and clinicopathologic characteristics

Characteristics	Number	n384546 expression		*p*-value
		Low	High	
Gender				
Male	34	16	18	0.839
Female	83	42	41	
Age				
≤45	49	24	25	1.000
>45	68	34	34	
Tumor size				
≤2 cm	91	52	39	0.003*
>2 cm	26	6	20	
Lymph node metastasis (VI)				
Yes	58	19	39	<0.001*
No	59	39	20	
Lymph node metastasis (II, III, IV)				
Yes	23	5	18	0.005*
No	94	53	41	
TNM stage				
I	84	47	37	0.039*
II–IV	33	11	22	

Low/high by the sample median. Pearson *χ*^2^ test

*p < 0.05 was considered statistically significant

### Effects of n384546 on PTC cell proliferation, apoptosis, migration, and invasion both in vitro and in vivo

**Fig. 2:**
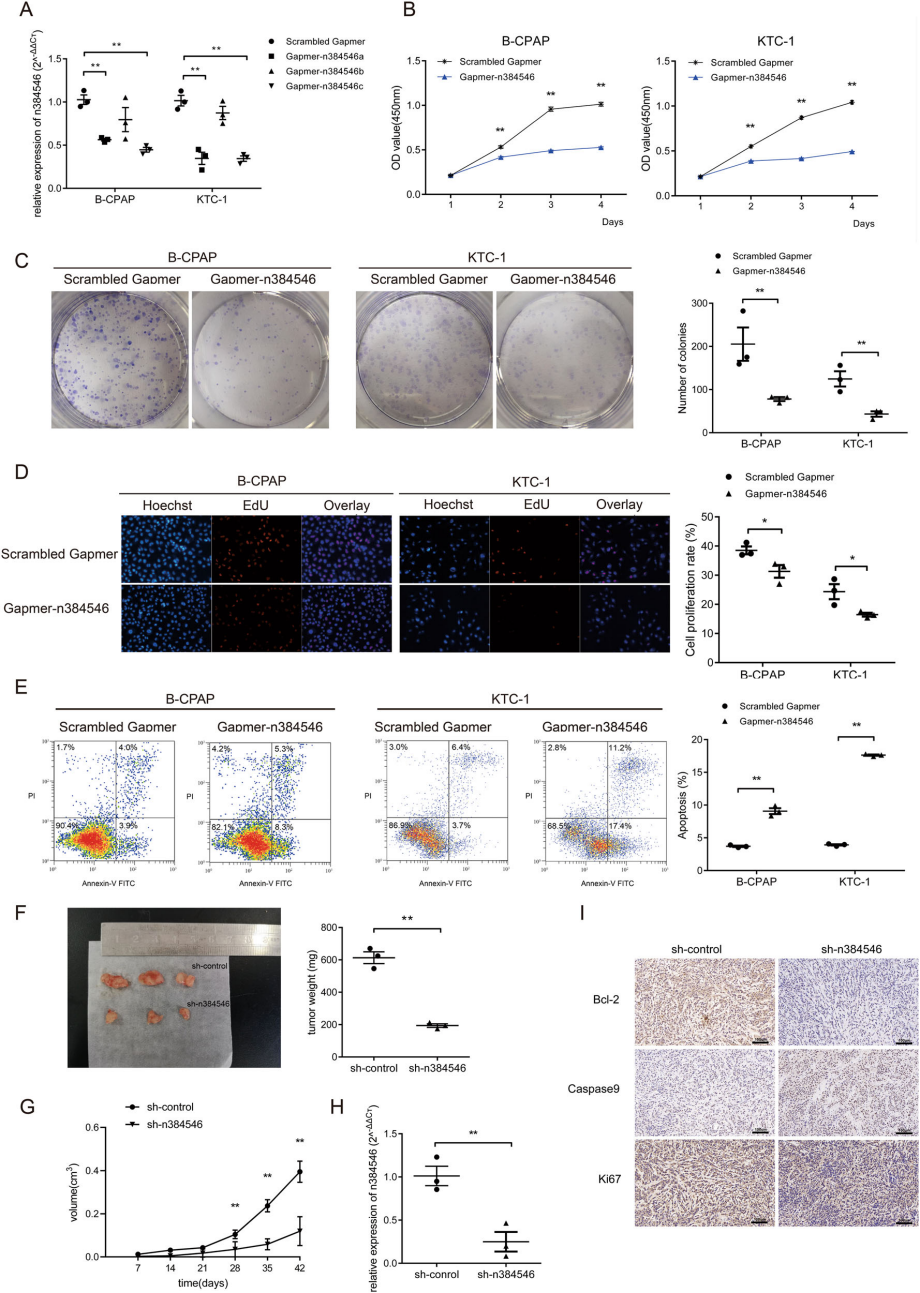
Effects of n384546 on thyroid papillary cancer cell proliferation, viability, and apoptosis in vitro and in vivo. **a** Validation of Gapmer-n384546 knockdown efficiency in B-CPAP and KTC-1 cells was determined by qRT-PCR. **b** CCK-8 proliferation assay in Scrambled Gapmer or Gapmer-n384546 transfected B-CPAP and KTC-1 cells. **c** Colony formation assay in Scrambled Gapmer or Gapmer-n384546 transfected B-CPAP and KTC-1 cells. **d** EdU proliferation assay in Scrambled Gapmer or Gapmer-n384546 transfected B-CPAP and KTC-1 cells. **e** Flow cytometric analysis of apoptosis in Scrambled Gapmer or Gapmer-n384546 transfected B-CPAP and KTC-1 cells. **f** Tumor size and tumor weight of nude mice was measured and analyzed. **g** Tumor volume curves of nude mice injected with sh-control and sh-n384546 B-CPAP cells were analyzed. **h** n384546 expression in tumors collected from nude mice was determined by qRT-PCR. **i** Immunohistochemical staining of Bcl-2, caspase9 and Ki-67 was used to assess proliferation and apoptosis (400×). Data represent the mean ± SEM of three separate experiments. All experiments were repeated at least three times. *p < 0.05, **p < 0.01 in independent Student’s *t* test

In order to determine the function of n384546 in PTC, we further investigated whether inhibition of n384546 could affect PTC cell biologic activity. LncRNA n384546 knockdown in B-CPAP and KTC-1 cell lines was achieved using Gapmer-n384546, and a Scrambled Gapmer served as the negative control, as shown in Fig. 2a. Gapmer-n384546c had the highest knockdown efficiency.

The effects of n384546 on PTC cell proliferation were measured by CCK8, EdU assays, and a colony formation assay. The CCK8 and EdU assays demonstrated that the proliferation and viability were decreased in Gapmer-n384546 transfected B-CPAP and KTC-1 cells compared with that in Scrambled Gapmer transfected cells. Colony formation assays showed that knockdown of n384546 significantly reduced colony formation capacity (Fig. 2b–d). We also detected a significantly increased percentage of apoptotic cells in Gapmer-n384546 transfected B-CPAP and KTC-1 cells compared with Scrambled Gapmer transfected cells by flow cytometry with Annexin V and PI double straining (Fig. 2e). To confirm whether n384546 promotes PTC tumorigenesis in vivo, we used a lentiviral shRNA system to knock down n384546 efficiently in B-CAP cells. Then, B-CPAP cells infected Lv-shn384546 and Lv-shNC were hypodermically injected into nude mice (*n* = 3 each group). Tumor growth was significantly inhibited in Lv-shn384546 infected cells compared with Lv-shNC infected cells (Fig. 2f, g). The expression level of n384546 was notably downregulated in tissues infected with Lv-shn384546 compare with Lv-shNC (Fig. 2h). In addition, IHC staining of resected tumor tissues showed the proliferation marker Ki67 was remarkably reduced in Lv-shn384546 cells compared with Lv-shNC cells. Moreover, the expression of bcl-2 was decreased in Lv-shn384546 cells while caspase9 expression was increased, which demonstrated that cell apoptotic activity was significantly increased after knockdown of n384546 (Fig. 2i).

**Fig. 3:**
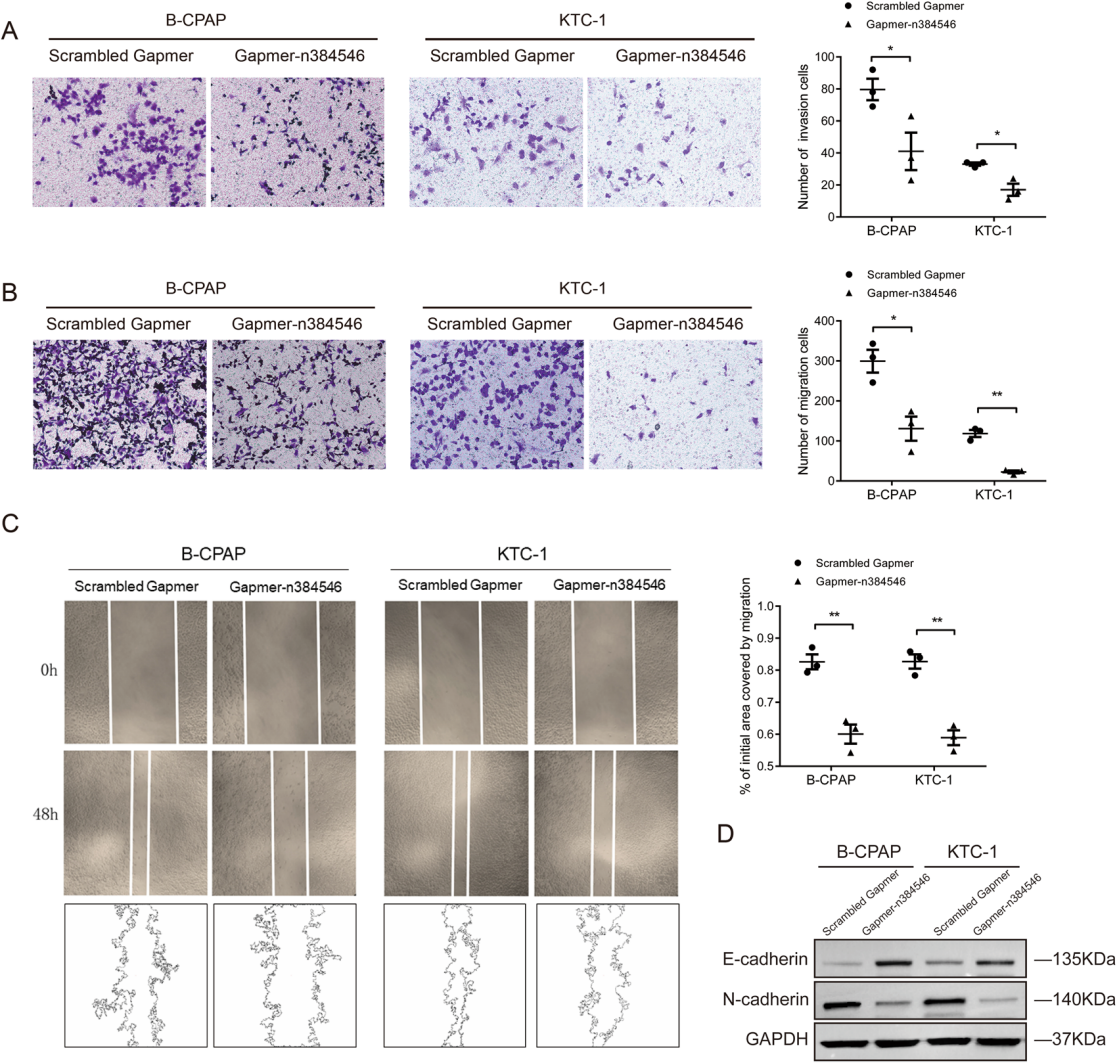
Effects of n384546 on thyroid papillary cancer cell migration and invasion. **a** Transwell migration assay in Scrambled Gapmer or Gapmer-n384546 transfected B-CPAP and KTC-1 cells. **b** Transwell invasion assay in Scrambled Gapmer or Gapmer-n384546 transfected B-CPAP and KTC-1 cells. **c** Wound healing assay in Scrambled Gapmer or Gapmer-n384546 transfected B-CPAP and KTC-1 cells. **d** Expression of E-cadherin and N-cadherin in Scrambled Gapmer or Gapmer-n384546 transfected B-CPAP and KTC-1 cells was determined by Western blot. Data represent the mean ± SEM of three separate experiments. All experiments were repeated at least three times. *p < 0.05, **p < 0.01 in independent Student’s *t* test (**a**–**c**)

In addition, transwell assays and wound healing assays were implemented to determine migration and invasion ability of PTC cells. As shown in Fig. 3a–c, after transfection of Gapmer-n384546, the migration and invasion abilities were both significantly inhibited in B-CPAP and KTC-1 cells compared with Scrambled Gapmer transfection. Moreover, western blot analysis showed that the protein level of E-cadherin was remarkably increased while N-cadherin was decreased in Gapmer-n384546 transfected cells compared with Scrambled Gapmer transfected cells. This indicated that EMT ability was inhibited after knockdown of n384546, which was consistent with the previous results (Fig. 3d).

### n384546 targeted and negatively regulated miR-145-5p

**Fig. 4:**
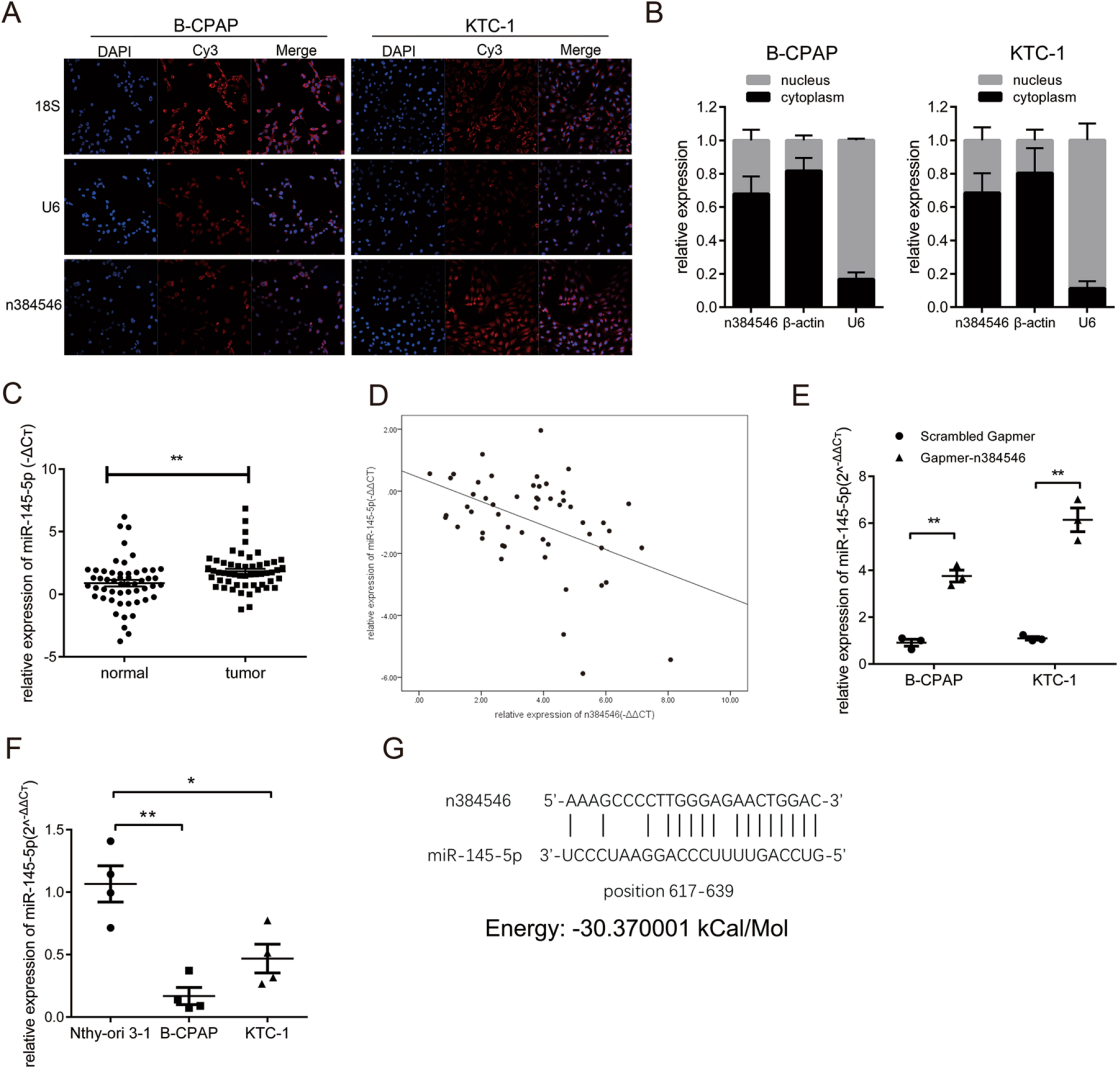
MiR-145-5p is regulated by n384546. **a** Localization of n384546 in B-CPAP and KTC-1 cells by fluorescent in situ hybridization. Nuclei are stained blue (DAPI) and n384546 is stained red. 18S is localized in the cytoplasm and U6 is localized in the nucleus. **b** The percentage of n384546, β-actin, and U6 in the cytoplasm and nucleus fraction of B-CPAP and KTC-1 cells was determined by qRT-PCR. **c** MiR-145-5p expression in 53 pair samples of PTC and adjacent normal tissues was determined by qRT-PCR. **d** Negative correlation between n384546 and miR-145-5p expression in PTC patients (Pearson Correlation Coefficient = −0.459, *p* < 0.01). **e** MiR-145-5p expression in Scrambled Gapmer or Gapmer-n384546 transfected B-CPAP and KTC-1 cells was determined by qRT-PCR. **f** Relative levels of miR-145-5p in normal thyroid cell Nthy-ori 3-1 and two types of PTC cells, B-CPAP and KTC-1 were determined by qRT-PCR. **g** The predicted binding sites and binding energy of miR-145-5p to the n384546 sequence. Data in (**d**) represent the mean ± SEM of three separate experiments. Data in (**e**) represent the mean ± SEM of four separate experiments. *p < 0.05, **p < 0.01 in paired Student’s *t* test (**b**) and independent Student’s *t* test (**d**, **e**)

**Fig. 5:**
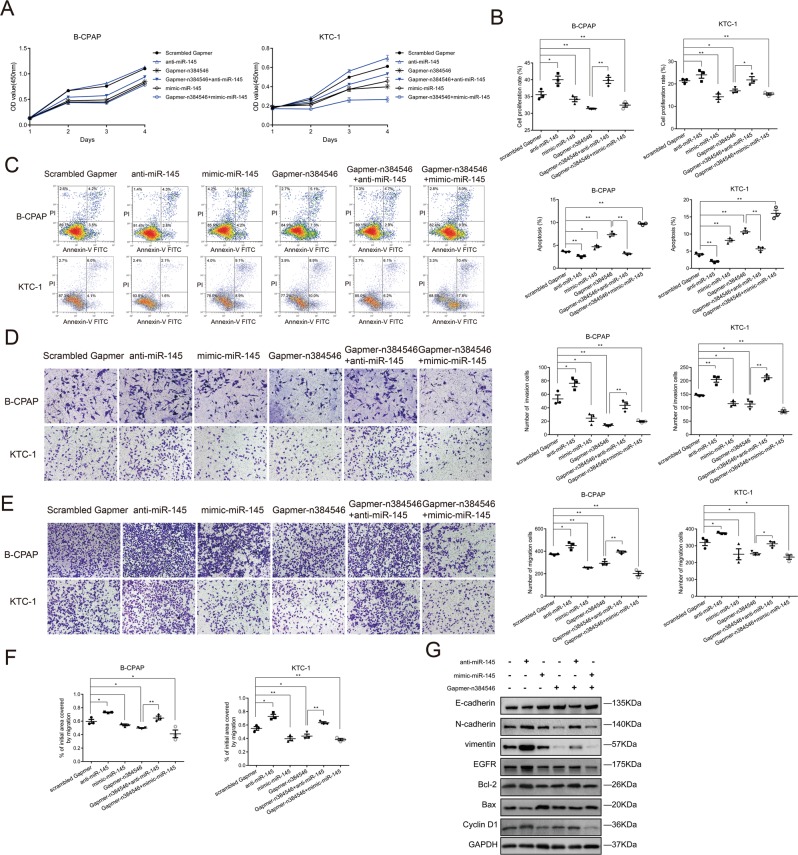
Anti-miR-145 reversed Gapmer-n384546 induced suppression of proliferation, apoptosis, migration, and invasion. **a** CCK-8 proliferation assay, **b** EdU proliferation assay, **c** Flow cytometric analysis of apoptosis, **d** Transwell invasion assay, **e** Transwell migration assay, and **f** Wound healing assay were performed in B-CPAP and KTC-1 cells transfected with Scrambled Gapmer, anti-miR-145, mimic-miR-145, Gapmer-n384546, Gapmer-n384546 + anti-miR-145, Gapmer-n384546 + mimic-miR-145. **g** The expression of proteins in B-CPAP cells transfected with Scrambled Gapmer, anti-miR-145, mimic-miR-145, Gapmer-n384546, Gapmer-n384546 + anti-miR-145, Gapmer-n384546 + mimic-miR-145 was determined by western blot. Data represent the mean ± SEM of three separate experiments. All experiments were repeated at least three times. *p < 0.05, **p < 0.01 in independent Student’s *t* test (**a**–**f**)

Then, we further investigated the underlying molecular mechanism by which n384546 regulates PTC cell proliferation and metastasis. Recently, accumulating evidence indicated that lncRNAs could act as competing endogenous RNA (ceRNA) in modulating the biological functions of miRNAs^6,17–19^. To examine whether n384546 could act as a ceRNA, we performed RNA-FISH and qRT-PCR to confirm the location of n384546 in PTC cells. As shown in Fig. 4a, b, n384546 was localized both in the cytoplasm and nucleus of B-CPAP and KTC-1 cells, which means it could function as a ceRNA. The potential miRNA targets of n384546 were predicted using bioinformatics databases such as RegRNA 2.0 (http://regrna2.mbc.nctu.edu.tw/) and miRANDA (http://www.microrna.org). We identified that miR-145-5p, miR-422a, and miR-505 may have putative binding sites with n384546. Previous studies also showed that downregulation of these miRNAs may serve as an independent prognosis factor in several cancers^20–24^. To further identify the miRNA target of n384546, we performed qRT-PCR to detect these three miRNAs in tissues from PTC patients. The results revealed that all miRNAs were downregulated in PTC tissues (Fig. 4c, Fig. S2A-B), but only the expression level of miR-145-5p in PTC patients was negatively correlated with n384546 (Fig. 4d, Fig. S2C-D). In addition, we observed that miR-145-5p was significantly upregulated in n384546 knockdown PTC cells, while miR-422a and miR-505 expression did not change (Fig. 4e, Fig. S2E,F). This confirmed that miR-145-5p was negatively modulated by n384546. Compared with Nthy-ori 3-1 cells, B-CPAP and KTC-1 cells also had lower expression of miR-145-5p (Fig. 4f). Furthermore, miranda software predicted the binding energy between n384546 and miR-145-5p is −30.370001 kCal/Mol, which strongly supported the hypothesis that n384546 plays the role as an oncogene by binding miR-145-5p (Fig. 4g)^25^. To characterize whether n384546 exerted its function through miR-145-5p in PTC cell lines, we co-transfected Gapmer-n384546 and anti-miR-145 or mimic-miR-145 into the B-CPAP and KTC-1 cells. As shown in Fig. 5, the proliferation, migration, and invasion abilities of Gapmer-n384546 or mimic-miR-145 transfected cells were decreased compared with Scrambled Gapmer transfected cells. Also, the percentage of apoptotic cells in Gapmer-n384546 or mimic-miR-145 transfected cells increased. These results revealed that mimic-miR-145 had the similar effect on proliferation, apoptosis, migration, and invasion of PTC cells as Gapmer-n384546. The inhibition of cell proliferation and viability induced by Gapmer-n384546 was partially reversed by anti-miR-145 co-transfection in both B-CPAP and KTC-1 cells (Fig. 5a, b, Fig. S3), and the effect of Gapmer-n384546 on cell apoptosis could also be reversed by anti-miR-145 (Fig. 5c). Transwell and wound healing assays showed suppression of migration and invasion abilities in PTC cells induced by Gapmer-n384546 were partially reversed by anti-miR-145 (Fig. 5d–f, Fig. S4). Western blot analysis showed that effect of Gapmer-n384546 on apoptosis-related proteins and EMT-related proteins could be reversed by anti-miR-145 (Fig. 5g). These results suggested that the function of n384546 in PTC partially rely on miR-145-5p.

### n384546 regulated AKT3 expression by sponging miR-145-5p

**Fig. 6:**
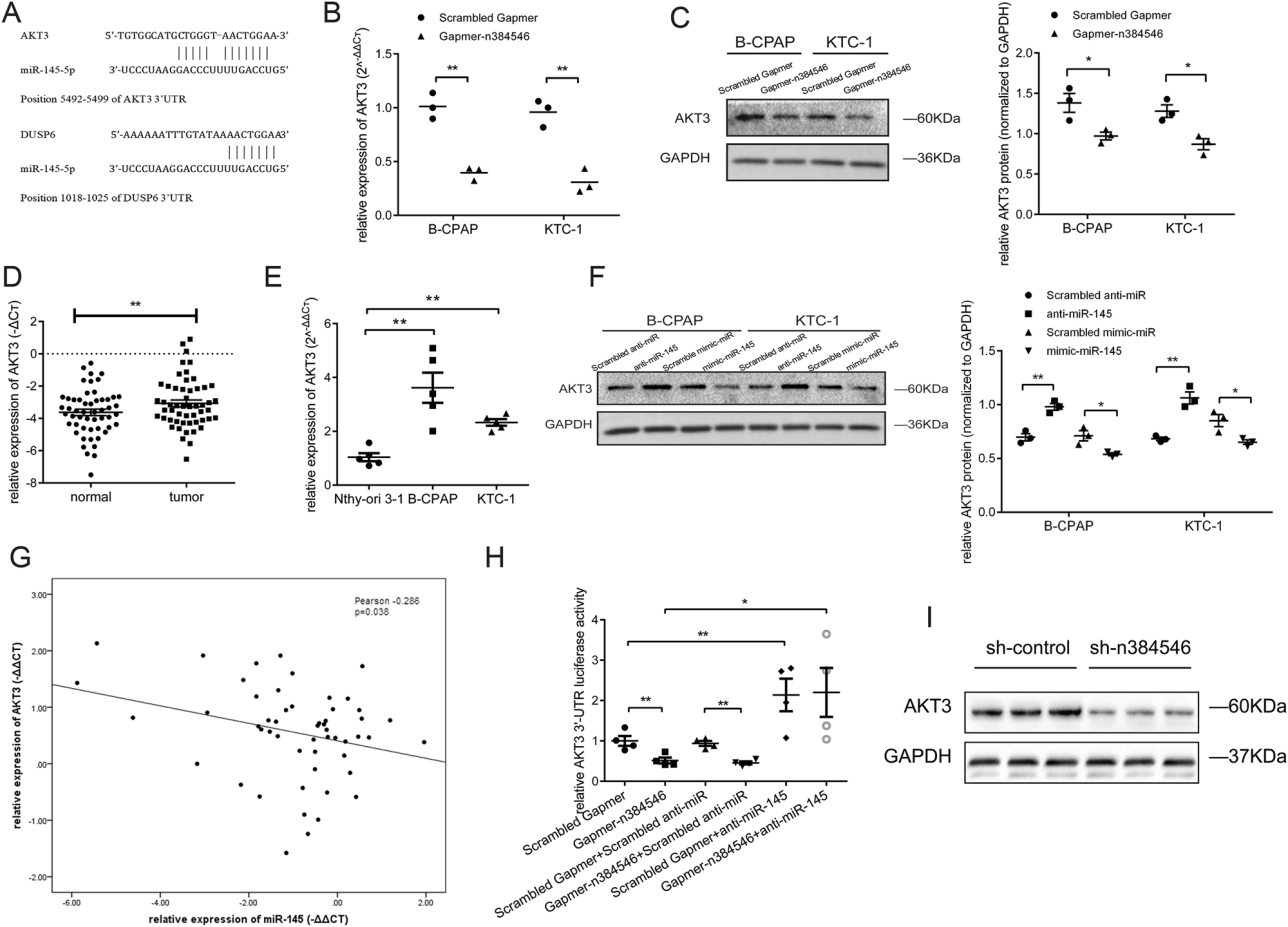
n384546 regulated AKT3 expression by sponging miR-145-5p. **a** The predicted binding sites of miR-145-5p to the AKT3 and DUSP6 sequence. **b**, **c** The AKT3 expression in Scrambled Gapmer or Gapmer-n384546 transfected B-CPAP and KTC-1 cells was determined by qRT-PCR and Western blot. **d** AKT3 expression in 53 pair samples of PTC and adjacent normal tissues. **e** Relative levels of AKT3 in normal thyroid cell Nthy-ori 3-1 and two types of PTC cells, B-CPAP and KTC-1, were determined by qRT-PCR. **f** The AKT3 expression in B-CPAP and KTC-1 cells transfected with scrambled anti-miR, anti-miR-145, scrambled mimic-miR, or mimic-miR-145 was determined by western blot. **g** Negative correlation between miR-145-5p and AKT3 expression in PTC patients (Pearson Correlation Coefficient = −0.286, *p* < 0.05). **h** Hela cells were co-transfected Scrambled Gapmer or Gapmer-n384546 and scrambled anti-miR or anti-miR-145. Luciferase activity was detected 24 h after transfection using the dual-luciferase assay. **i** AKT3 expression in tumors collected from nude mice was determined by western blot. Data in (**b**), (**c**), (**f**) represent the mean ± SEM of three separate experiments. Data in (**e**) represent the mean ± SEM of five separate experiments. Data in (**h**) represent the mean ± SEM of four separate experiments. All experiments were repeated at least three times. *p < 0.05, **p < 0.01 in paired Student’s *t* test (**d**) and independent Student’s *t* test (**b**, **c**, **e**, **f**, **h**)

In view of these results, we tried to predicted possible target genes of miR-145-5p. A previous study showed Akt signaling was inhibited after miR-145 overexpression in thyroid cancer cells, while AKT3 is a target of miR-145^21^. Another study reported that overexpression of miR-145 could inhibit cell proliferation by targeting DUSP6 in thyroid cancer^20^. By using the Targetscan database and miRanda algorithms, we found that 3′UTR regions of AKT3 and DUSP6 both have the predicted binding sites of miR-145-5p (Fig. 6a). This result is consistent with previous reports that AKT3 and DUSP6 are target genes of miR-145-5p^21,24^. We further examined the expression levels of AKT3 and DUSP6 in Scrambled Gapmer and Gapmer-n384546 transfected cells. Transfection of Gapmer-n384546 significantly decreased AKT3 expression in both mRNA and protein levels compared with Scrambled Gapmer (Fig. 6b, c). But the expression of DUSP6 did not change after Gapmer-n384546 transfection (Fig. S5). Furthermore, qRT-PCR analysis showed that AKT3 was significantly upregulated in the PTC specimens compared with normal specimens in PTC patients (Fig. 6d). In addition, AKT3 expression was higher in PTC cells compared with Nthy-ori 3-1 cells (Fig. 6e).

To verify whether miR-145-5p regulated AKT3, PTC cells were transfected with mimic-miR-145 or anti-miR-145 to increase or decrease miR-145-5p expression respectively. Results from western blot demonstrated that overexpression of miR-145-5p by mimic-miR-145 significantly decrease the level of AKT3 compared with Scrambled mimic-miR and conversely AKT3 expression significantly increased after transfected with anti-miR-145 compared with Scrambled anti-miR (Fig. 6f). The expression of AKT3 in PTC tissues was negatively associated with the expression of miR-145-5p by Pearson correlation analysis (Fig. 6g). Our results are consistent with previous research, which proved that miR-145-5p binds to the AKT3 transcript by luciferase reporter assay^21^.

Then, we used a Dual-luciferase Reporter Assay to verify that n384546 regulates AKT3 expression by sponging miR-145-5p. As shown in Fig. 6h, transfection of Gapmer-n384546 could significantly reduce the luciferase activity of AKT3 3′UTR compared with Scrambled Gapmer. The Gapmer-n384546 induced loss of AKT3 could efficiently be reversed by co-transfection of anti-miR-145. However, the upregulation of AKT3 3′UTR luciferase activity induced by anti-miR-145 could not be reversed by co-transfection of Gapmer-n384546. These results indicated that knockdown of n384546 could not decrease the AKT3 activity after inhibition of miR-145-5p, which confirmed that n384546 regulated the expression and activity of AKT3 by sponging miR-145-5p.

Furthermore, xenograft tumors from n384546 knockdown cells showed lower AKT3 expression compared to control cells (Fig. 6i), which demonstrated n384546 could regulate AKT3 expression in vivo.

## Discussion

Papillary thyroid carcinoma (PTC) is the most prevalent thyroid malignant tumor in clinical practice. However, the cause of PTC has not yet been entirely clear. Factors including family genetic, genetic mutations and radiation may cause tumors. In recent years, great progress has been made in research of molecular pathways involved in the progression of PTC such as *BRAF, RAS, TERT*, and *TP53*.

LncRNAs have been demonstrated to play key roles in several tumor biologic processes such as cell proliferation, apoptosis, and tumorigenesis^9–11,26^. Recent studies have shown that lncRNAs are dysregulated in PTC. For example, NAMA was downregulated in PTC with BRAF mutation and associated with growth arrest^27^. Transcripts of PTCSC2 are downregulated in PTC tissues, PTCSC2 is involved in the regulation and interaction of coding and noncoding genes in PTC cells^28^.

In the present study, RNA-seq was performed in two groups of PTC tissues, and a total of 86 lncRNA transcripts were differentially expressed in tumor vs. normal samples in both groups. Then a series of screen methods were used to determine candidate lncRNAs, and a novel lncRNA, n384546 may be a prognostic marker for PTC patients. Our RNA-seq results revealed that n384546 level was upregulated in PTC tissues compared to adjacent normal thyroid tissue. Further investigation verified that n384546 was significantly highly expressed in PTC tissues, while PTC patients with larger tumor size, positive lymph nodes metastasis or higher TNM stage showed more upregulated n384546 expression than patients with smaller tumor size, negative lymph nodes metastasis or lower TNM stage, which suggested that n384546 expression is involved in the development of PTC. In an in vitro model, downregulation of n384546 by Gapmer-n384546 inhibited cell proliferation, migration, and invasion, and promoted cell apoptosis. In an in vivo model, we used a lentiviral shRNA system to knock down n384546 stably in B-CPAP cells and found tumor growth was significantly inhibited in nude mice following subcutaneous injection of B-CPAP cells infected with Lv-shn384546. These results confirmed the oncogenic role of n384546 and identified n384546 is a positive regulator in the progression of PTC.

Then, we further investigated the underlying mechanism by which n384546 promoted the tumorigenesis process of PTC. Recently, the ceRNA hypothesis has attracted growing attention. Several studies have reported that lncRNAs could act as ceRNAs by competitively binding to miRNAs and thus modulating the expression of miRNA downstream targets. For instance, in breast cancer, H19 differentially sponges miR-200b/c and let-7b to mediate EMT and MET plasticity^29^. In addition, lncRNA-MIAT upregulates the level of VEGF, which plays an important role in pathological angiogenesis, by sequestering miR-150-5p and then regulate endothelial cell function^19^. Based on our bioinformatics analysis, we found that miR-145-5p may have putative binding sites with n384546. Several studies have demonstrated that the expression of miR-145^30^ is significantly decreased in various tumors including thyroid cancer^21,31–33^. In our study, we found that there was an inverse correlation between the expression of n384546 and miR-145-5p in PTC tissues. After transfection with Gapmer-n384546, miR-145-5p was significantly upregulated in PTC cell lines. The function of mimic-miR-145 in PTC cell proliferation and metastasis was similar to Gapmer-n384546. And anti-miR-145 could partially reverse the consequents of transfection of Gapmer-n384546. We further explored whether n384546 functions by affecting distribution of miR-145-5p on its specific targets. Bioinformatics analysis predicted that AKT3 is a potential target of miR-145-5p, which was confirmed in previous studies^21,34^. The PI3K/Akt pathway plays an important role in cell proliferation, EMT, cell cycle, and apoptosis in various cancers^35–37^. AKT, which is a key protein in many signaling pathway, can inhibit apoptosis and promote proliferation by affecting the activation status of various downstream factors^38^. Numerous studies have shown that aberrant AKT/PKB signaling pathway can lead to excessive cell proliferation and inhibition of apoptosis, which is intimately related to the occurrence and development of many malignant tumors^39^. At present, most studies focus on AKT1 and AKT2. AKT1 is frequently overexpressed in malignant tumors such as gastric cancer and lung cancer, and is associated with sustained proliferation of tumor cells^40^. AKT2 is mainly found in pancreatic cancer, ovarian cancer, and breast cancer, and is related to the continued survival of tumor cells^41^. It is reported that AKT3 is overexpressed in glioma, melanoma, and ovarian cancer. AKT3 participates in cell proliferation, inhibition of apoptosis and invasion, and metastasis of malignant tumors^42^. For thyroid cancer, AKT3 was upregulated in thyroid cancer tissues and cells as well^43^. But, the biological function of AKT3 and its role in the progression of PTC remain unknown. In our present research, miR-145-5p upregulation could suppress the expression of AKT3, while downregulation of miR-145-5p led to an increase in AKT3 expression. Our design for the luciferase reporter assay was novel, and it indicated that Gapmer-n384546 could reduce the luciferase activity of 3′-UTR of AKT3, which was restored after treatment with anti-miR-145. This indicated that n384546 regulates the expression of AKT3 by miR-145-5p. Moreover, we found that n384546 knockdown could also inhibit AKT3 expression both in vitro and in vivo. These consequences suggested that the effects of n384546 on PTC progression and metastasis could be partially attributed to sponging miR-145-5p and regulating AKT3 expression.

However, there are still limitations in our study. For example, the high-throughput RNA sequencing was performed in only two groups of samples, so this limited further bioinformatics analysis and the search for other mechanisms of n384546 in PTC progression. Also, we did not study whether inhibition of miR-145-5p could abolished the function of n384546 knockdown in vivo. In future studies, we will examine the effects of miR-145-5p inhibition in n384546 knockdown cells in vivo and investigate other mechanisms by which n384546 plays the role as an oncogene in PTC.

To sum up, we show that the novel lncRNA n384546 is highly expressed in PTC tissues and cell lines. Overexpression of n384546 was correlated with large tumor size, lymph node metastasis, and TNM stage. Knockdown of n384546 suppressed the progression and metastasis of PTC cells both in vitro and in vivo. Our study indicates that n384546 exerts its oncogenic properties in PTC tumorigenesis by sponging miR-145-5p and then regulating its target AKT3, which has been proven to be an oncogene in several cancers. LncRNA n384546 might be a key regulator of PTC cell progression and metastasis, and may be a potential biomarker for PTC diagnosis and treatment.

## Materials and Methods

### Human specimens

Human tissues were collected from 69 patients who underwent standard surgical procedures between January 2015 and December 2016 at the Department of Head and Neck Surgery, Renji Hospital, School of Medicine, Shanghai Jiaotong University (P.R. China). RNA-seq was conducted on 16 pairs of tissues, and qRT-PCR validation was performed on the other 53 pairs. The patient information is shown in Table S3. Patients were excluded if they received non-surgical treatment such as chemotherapy, radiotherapy, or molecular targeted therapy prior to surgery. The use of human PTC tissue specimens in this study was approved by the Ethics Committee of Renji Hospital, School of Medicine, Shanghai Jiaotong University (Shanghai, China), and written informed consent was obtained from all patients or their guardians before sample collection.

### Cell lines

The human normal thyroid epithelial cell line (Nthy-ori 3-1) was purchased from the American Type Culture Collection (ATCC, USA). Nthy-ori 3-1 cells were cultured in F12K medium (Gibco, USA) containing 20% FBS (Gibco, USA). And the human papillary thyroid carcinoma cell lines (B-CPAP, KTC-1) were kindly provided by Stem Cell Bank, Chinese Academy of Sciences. B-CPAP and KTC-1 cells were cultured in RPMI-1640 medium (Gibco, USA) containing 10% FBS. All cells were cultured at 37 °C in incubator with 95% air and 5% CO_2_. All cell lines were detected for Mycoplasma before use, and the identity of all cell lines was verified by short tandem repeat (STR) analysis in 2017.

### High-throughput RNA sequencing and analysis of RNA-seq data

Sixteen PTC patients were randomly divided into two groups. The cancer tissues and adjacent normal tissues of eight patients in the same group were mixed together in equal amounts separately, each sample representing eight individual patients. Total RNA of the four samples was extracted using TRIzol (Invitrogen, USA). Only RNA with RIN (RNA integrity number) >7 was used for further study. RNA was fragmented into short fragments and reverse transcribed to cDNA library. Finally, high-throughput RNA-seq was carried out using the platform Illumina HiSeqTM 2000.

Mapping and alignment RNA-seq reads was performed using the human genome version hg38 GRCh38, the total raw reads of each sample ranged from 47 to 48 million. Raw reads from each sample was assembled and predicted separately to obtain a potential novel lncRNA, then the results of multiple samples were clustered and de-redundant, and the assembly results were optimized^44^. Quantitative and differential expression analysis of the predicted lncRNAs was performed. The transcript expression was counted by FPKM (fragments per kilobase of exon model per million mapped reads). FDR (false discovery rate) <0.05 and fold change >2 times were used as criteria to screen differentially expressed transcripts by Cuffdiff software. The Gene Expression Omnibus accession numbers for the RNA-seq data for PTC tissues is GSE124841.

### RNA extraction, reverse transcription, and qRT-PCR

Total RNA of cells and tissues was extracted using TRIzol reagent and reverse transcribed into cDNA using the PrimerScript™ reagent Kit (TaKaRa, Dalian, China). qRT-PCR experiments were conducted with the SYBR Premix Ex Taq (TaKaRa, Dalian, China) on the ABI StepOne™ Real-Time PCR system (Applied Biosystems, CA). The quantification of miRNAs was detected using miScript SYBR Green PCR Kit (Qiagen, Hilden, Germany). Relative expression values of genes were analyzed by the 2^−ΔΔCt^ method using β-actin and U6 for standardization. The primer sequences used for qRT-PCR are listed in Table S1.

### Silencing of lncRNA

LNA™ longRNA GapmeRs (Exiqon, Denmark) are antisense oligonucleotides with perfect sequence complementary to their RNA target. The 2′C and 4′C atoms of these modified nucleotideare are linked by an oxymethylene bridge, the affinity of the LNA for target sequences increases and off-target effect significantly decreased^45,46^.

GapmeRs against n384546 were used for posttranscriptional lncRNA silencing. The sequences are shown in Table S3. Cells were transfected with 50 µM LNA longRNA GapmeRs with Lipofectamine 2000 reagent (Invitrogen, USA).

### Silencing and overexpression of miRNA

Anti-miR-145-5p and mimic-miR-145-5p were purchased from RiboBio (Guangzhou, China). Cells were transfected with 50 µM mimic-miR or 100 µM anti-miR with Lipofectamine 2000 reagent.

### In vivo experiment

Nude mice (BALB/c nude; 4 weeks old) were purchased from the Experimental Animal Centre of Shanghai Institutes for Biological Sciences and kept under pathogen-free conditions. In order to identify the effect of n384546 on tumor growth, B-CPAP cells infected Lv-shn384546 and Lv-shNC stably were collected (5 × 10^7^ cells in 200 μl) and subcutaneously injected into on the right side of axillary. The shRNA sequences are shown in Table S3. Tumor size and weight were measured every 7 days. After monitoring the tumor size for 42 days, the mice were euthanized and the tumor nodes were collected to assess tumor size. The excised tissues were frozen in liquid nitrogen for further analysis. All animal studies were approved by the Shanghai Jiao Tong University Affiliated Sixth People’s Hospital Animal Care and Use Committee (Shanghai, China).

### Fluorescent in situ hybridization (FISH) for detection of n384546

B-CPAP and KTC-1 cells were fixed and then permeabilized with 0.5% Triton X-100. CY3-labeled probe for n384546 (Ribo, China) at 5 ng/μl concentration were added and overnight hybridization reaction was performed at 37 °C. Then, the slides of cells were stained with DAPI, after that a confocal microscopy (Zeiss LSM 710) was used to photo the cells.

### Colony formation assay

B-CPAP and KTC-1 cells were seeded at a density of 200 cells/well in 6-well cultural plates 24 h after transfection and then cultured in complete growth medium. After about 10 days, the cells were fixed and then stained with giemsa. The stained cell colonies were imaged and counted by Image-Pro Plus software.

### EdU and CCK8 assay for cell proliferation

The EdU (5-ethynyl-2′-deoxyuridine) assay was conducted using Cell-Light EdU In Vitro Imaging Kit (Ribo, China). In brief, 2000–5000 cells were seeded into 96-well cultural plates. After 24 h, EdU dissolved in medium was added into each well and then incubated for about 2 h at 37 °C. Then the cells were fixed and stained with Hoechst33342 and Apollo reaction mixture. Images were photographed using fluorescence microscopy and cells were counted using Image-Pro Plus software.

Cell proliferation and viability were analyzed using CCK8 (Cell Counting Kit-8, Dojindo, Japan) following the protocol provided by the manufacturer. Twenty-four hours after transfection, cells were seeded in 96-well cultural plates at a density of 2000 cells/well. Then 10 μl CCK8 solution was added into each well. After incubation for 2 h with CCK8, the absorbance of all wells was recorded by an automated plate reader at 450 nm.

### Wound healing, migration, and invasion assays

For the wound healing assay, B-CPAP and KTC-1 cells were seeded in 6-well cultural plates and then transfected with either Gapmer-n384546 or Scrambled Gapmer after reaching 80% confluency. Twenty-four hours after transfection, a 200-μl pipette tip was used to create wound gaps. Cells were washed and cultured in serum-free medium. The wound gaps were observed and photographed at 0, 12, 24, and 48 h after wounding.

For transwell migration assay, cells (1 × 10^5^) were suspended in 200 µl serum-free medium and then added into the upper chambers of 8.0 mm transwell chambers (Corning, USA). For transwell invasion assay, cells (2 × 10^5^) were added into the upper chambers pre-coated with diluted Matrigel (1:5, BD Biosciences, USA) on their membrane. Then, the transwell chambers were put into 24-well plates containing medium with 10% FBS in the lower chambers. After 24 h, the cells which pass through the membrane were fixed and stained with crystal violet. The stained cells were photographed using a microscope and counted using Image-Pro Plus software.

### Flow cytometric analysis of cell apoptosis

Annexin V-FITC/PI kits (BD Biosciences, USA) were used to analyze cell apoptosis. In brief, forty-eight hours after transfection, cells were suspended with 100 μl Binding Buffer at a concentration of 1 × 10^5^ cells/ml. Then, 5 μl of FITC Annexin V and 5 μl of PI was added into the solution. After incubating for 15 min at room temperature protected from light, 400 μl Binding Buffer was added and cell apoptosis were examined using flow cytometry (BD FACS Calibur) within 1 h.

### Western blot analysis

Protein of tissues and cells was extracted using RIPA buffer with PMSF (Beyotime Biotechnology, Beijing, China). After measuring protein concentration, equal quantities of protein per sample were segregated using SDS–PAGE and gel electrophoresis was used to transfer the protein onto a nitrocellulose membrane. After blocking for 1 h, the membranes were incubated with primary antibodies at 4 °C overnight (Table S2). Then the membranes were incubated with secondary antibodies (Santa Cruz Biotechnology). The protein strips were visualized and detected using a chemiluminescence reagent (ECL) kit (Beyotime Biotechnology).

### luciferase activity assay

3′UTR reporter assay was performed in HeLa cells with pMIRREPORT b-galactosidase as the normalizer as we have described previously^47^. The plasmid-huAKT3 3′UTR (3′-untranslated region) region containing putative binding sites for miR-145-5p was generated using molecular cloning techniques and inserted into a pMIR-REPORT luciferase plasmid (Ambion). The primers are: AATTAAACTAGTCCTTTAGTGTTTGTCATTCTCAG (sense); ATCAGAAAGCTTGAGCTATTGAAACTTACTTTTTATTA (antisense).

### Statistical analysis

All experimental data were analyzed using SPSS 21.0 statistical analysis software (IBM, USA) and GraphPad Prism 5 software (GraphPad, USA) for statistical analysis. The differences between groups were estimated by two-tail Student’s *t* test. The association between lncRNA n384546 expression and clinicopathological features of PTC patients was analyzed by *χ*^2^ (Chi-square) test. Pearson correlation analysis was performed to detect the correlation between lncRNA and miRNA. A *p* value of <0.05 was considered statistically significant.

## Supplementary information

Supplementary Figure Legends

Supplementary Table 1

Supplementary Table 2

Supplementary Table 3

Supplementary Figure 1

Supplementary Figure 2

Supplementary Figure 3

Supplementary Figure 4

Supplementary Figure 5
