# Supplementary material for: A novel lncRNA n384546 promotes thyroid papillary cancer progression and metastasis by acting as a competing endogenous RNA of miR-145-5p to regulate AKT3
Source: Cell Death Dis. 2019 Jun 3;10(6):433. doi: 10.1038/s41419-019-1637-7 (PMC6547665; doi:10.1038/s41419-019-1637-7)
Supplement: Supplementary file 1 — Supplementary Figure Legends [file 41419_2019_1637_MOESM1_ESM.docx]

**Supplementary Figure Legends**

**Figure S1.** Information of n384546 in noncode.

**Figure S2.** miR-422a and miR-505 expression in PTC tissues and cells. (A) MiR-422a expression in 53 pair samples of PTC and adjacent normal tissues was determined by QRT-PCR (p<0.01). (B) MiR-505 expression in 53 pair samples of PTC and adjacent normal tissues was determined by QRT-PCR (p<0.01). (C) There is no correlation between n384546 and miR-422a expression in PTC patients (p=0.467). (D) There is no correlation between n384546 and miR-505 expression in PTC patients (p=0.372). (E) MiR-422a expression in scrambled Gapmer or Gapmer-n384546 transfected B-CPAP and KTC-1 cells was determined by QRT-PCR (B-CPAP p=0.854, KTC-1 p=0.740). (F) MiR-505 expression in scrambled Gapmer or Gapmer-n384546 transfected B-CPAP and KTC-1 cells was determined by QRT-PCR (B-CPAP p=0.734, KTC-1 p=0.654).

**Figure S3.** The EdU assay in B-CPAP and KTC-1 cells transfected with scrambled Gapmer, anti-miR-145, mimic-miR-145, Gapmer-n384546, Gapmer-n384546+anti-miR-145, Gapmer-n384546+mimic-miR-145.

**Figure S4.** The wound healing assay in B-CPAP and KTC-1 cells transfected with scrambled Gapmer, anti-miR-145, mimic-miR-145, Gapmer-n384546, Gapmer-n384546+anti-miR-145, Gapmer-n384546+mimic-miR-145.

**Figure S5.** DUSP6 expression in scrambled Gapmer or Gapmer-n384546 transfected B-CPAP and KTC-1 cells was determined by QRT-PCR (A) and western blot (B).
