# Supplementary material for: A novel lncRNA n384546 promotes thyroid papillary cancer progression and metastasis by acting as a competing endogenous RNA of miR-145-5p to regulate AKT3
Source: Cell Death Dis. 2019 Jun 3;10(6):433. doi: 10.1038/s41419-019-1637-7 (PMC6547665; doi:10.1038/s41419-019-1637-7)
Supplement: Supplementary file 2 — Supplementary Table 1 [file 41419_2019_1637_MOESM2_ESM.docx]

Table S1 Primer Sequence

| Gene Name |  | Primer Sequence (5' -> 3') |
| --- | --- | --- |
| β-actin | Forward: | CATGTACGTTGCTATCCAGGC |
|  | Reverse: | CTCCTTAATGTCACGCACGAT |
| n365626 | Forward: | TGGAGCCACATGAAGGAAGT |
|  | Reverse: | TTTGTGCAACGTGGAGAACC |
| n378806 | Forward: | TTCCTGGATTGAAGCCTGAC |
|  | Reverse: | TGGCCCTTCACCTAAATGAC |
| n374322 | Forward: | CCTGTCAAGATCCCATTGGC |
|  | Reverse: | GCCTCCTATACCCTCGGAAC |
| n384546 | Forward: | CCCTTCCACTCCCAGATTGT |
|  | Reverse: | AAGTCCAGTTCTCCCAAGGG |
| n378530 | Forward: | TTTCCTGCTTTATGGCGAAG |
|  | Reverse: | CTTGAACCCAGGAAGCAGAG |
| n343066 | Forward: | TCCTGAACACCTTAGGCTGG |
|  | Reverse: | TGATGTTGGGCTGATGAGGT |
| XLOC_007852 | Forward: | GAAGGAAGTGGGCATTGTGT |
|  | Reverse: | TGAATTTTGGGTGGAGAAGG |
| XLOC_007866 | Forward: | AGCATCTCCTTGCGTTTTTC |
|  | Reverse: | TGGTTTAAGCCAATCAGCAA |
| XLOC_012168 | Forward: | TCTACCATTCCCTTCCCTTG |
|  | Reverse: | TTCTACCACAACCACTGCTCA |
| XLOC_015463 | Forward: | CAGGGTTGGAAAGCACCTAA |
|  | Reverse: | TTGTTTGTGTTCCCTGCTCA |
| XLOC_021360 | Forward: | GAATGGGAGAAAACGCAAAA |
|  | Reverse: | AAAGGTTTTCGCCTCCTGTT |
| XLOC_035574 | Forward: | GAAATGGGGCTAGCACAGAA |
|  | Reverse: | AAGCCCCAAGAGTTGATCCT |
| XLOC_044506 | Forward: | GGGCTTACGTCCAGTGTTGT |
|  | Reverse: | TGGAGAAGGGAGGAATGATG |
| XLOC_045963 | Forward: | TCTGGGCAAGGGTAGAGAGA |
|  | Reverse: | GAGGGAAACCGGAGTAGGTC |
| AKT3 | Forward: | TGTGGATTTACCTTATCCCCTCA |
|  | Reverse: | GTTTGGCTTTGGTCGTTCTGT |
