# Supplementary material for: A novel lncRNA n384546 promotes thyroid papillary cancer progression and metastasis by acting as a competing endogenous RNA of miR-145-5p to regulate AKT3
Source: Cell Death Dis. 2019 Jun 3;10(6):433. doi: 10.1038/s41419-019-1637-7 (PMC6547665; doi:10.1038/s41419-019-1637-7)
Supplement: Supplementary file 3 — Supplementary Table 2 [file 41419_2019_1637_MOESM3_ESM.docx]

| **Antibody** | **Company** | **Reference ID** |
| --- | --- | --- |
| AKT3  DUSP6 | Cell signaling  Cell signaling | 3788  39441 |
| E-cadherin | Cell signaling | 14472 |
| N-cadherin | Cell signaling | 13116 |
| vimentin | Cell signaling | 49636 |
| EGFR | Cell signaling | 4267 |
| Bcl-2 | Cell signaling | 4223 |
| Bax | Cell signaling | 5023 |
| CyclinD1 | Cell signaling | 2978 |
| GAPDH | Affnity | AF7021 |
