# Supplementary figures and images for: A novel lncRNA n384546 promotes thyroid papillary cancer progression and metastasis by acting as a competing endogenous RNA of miR-145-5p to regulate AKT3
Source: Cell Death Dis. 2019 Jun 3;10(6):433. doi: 10.1038/s41419-019-1637-7 (PMC6547665; doi:10.1038/s41419-019-1637-7)

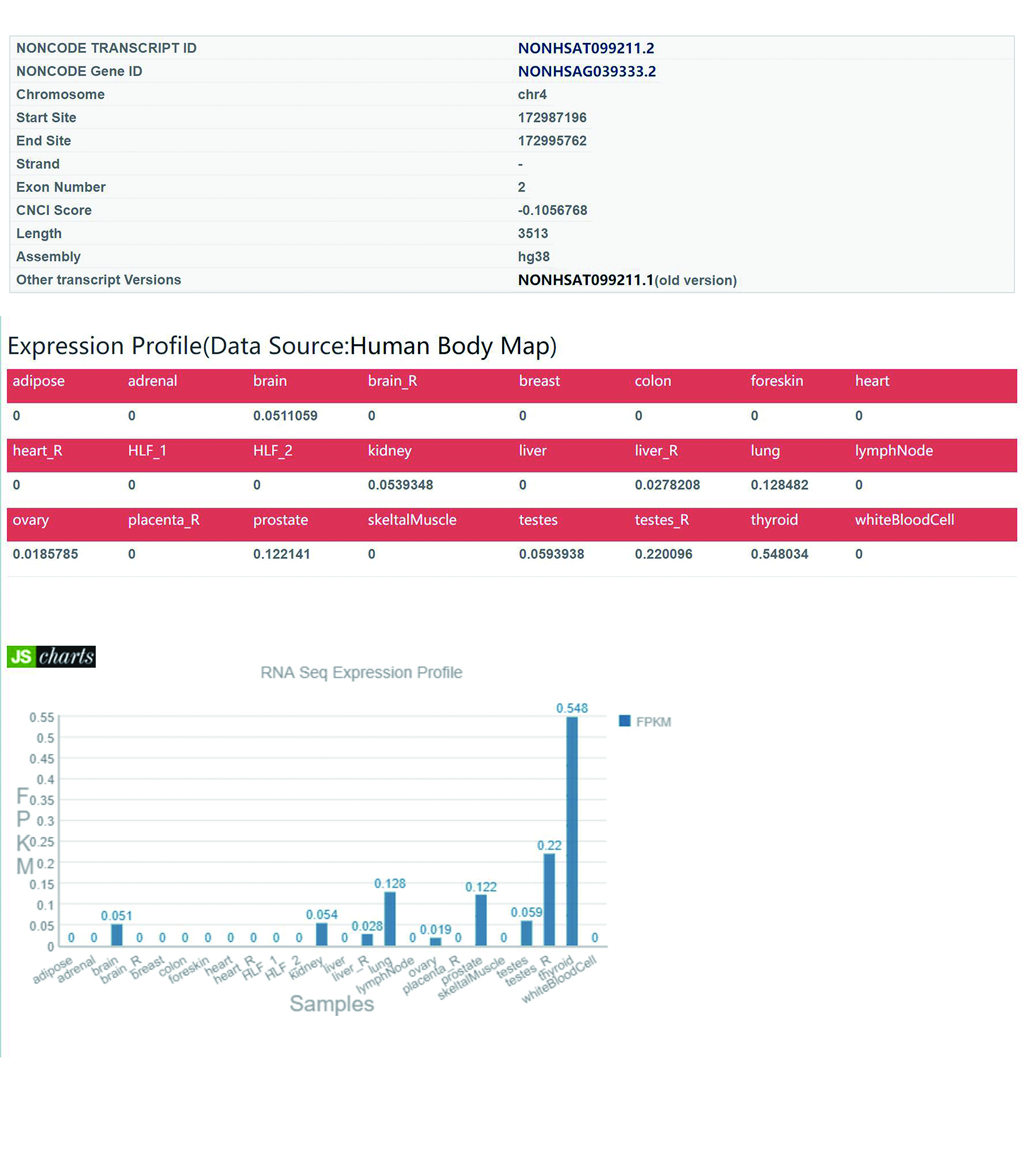

Supplement: Supplementary file 5 — Supplementary Figure 1 [file 41419_2019_1637_MOESM5_ESM.tif]

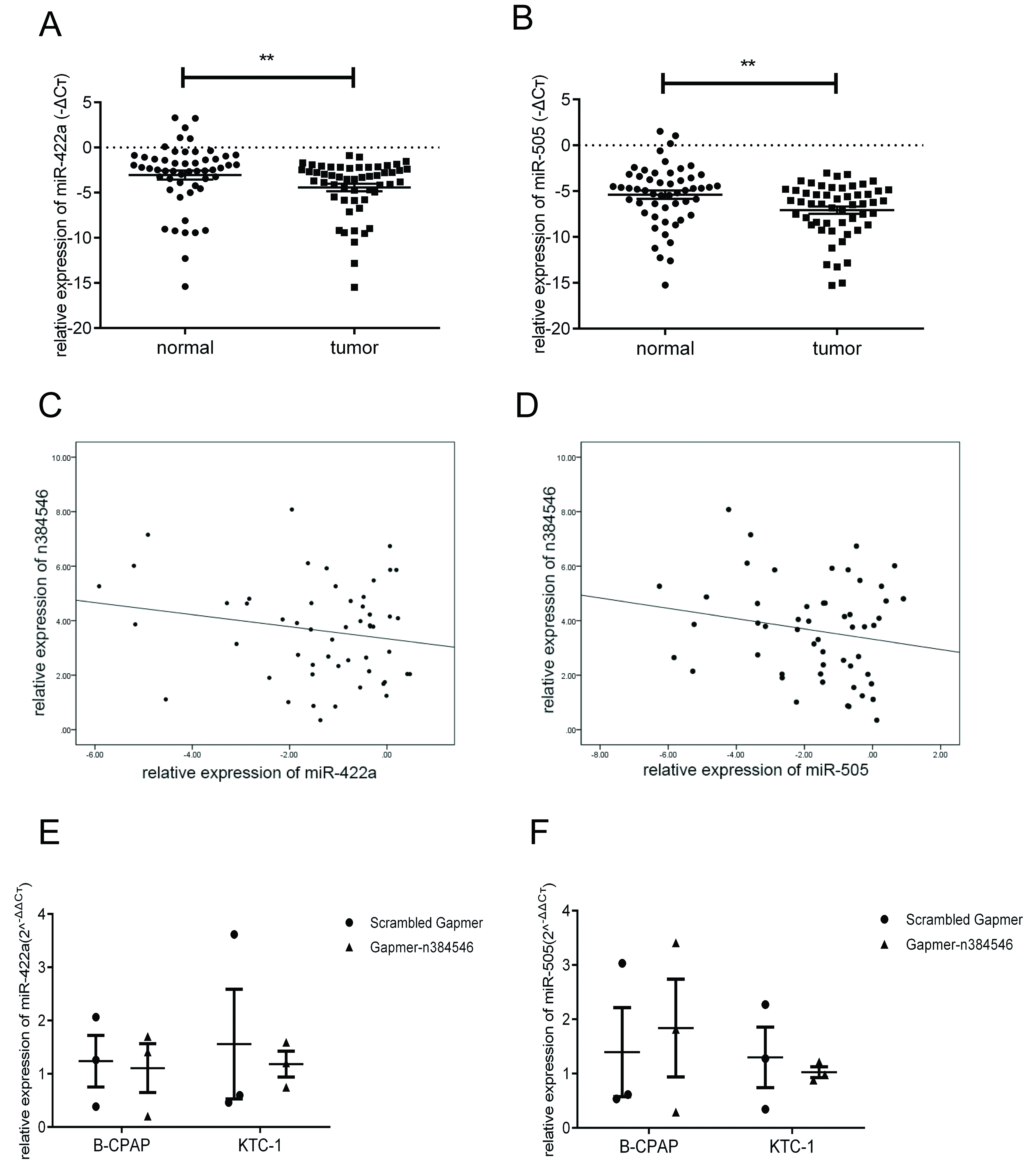

Supplement: Supplementary file 6 — Supplementary Figure 2 [file 41419_2019_1637_MOESM6_ESM.tif]

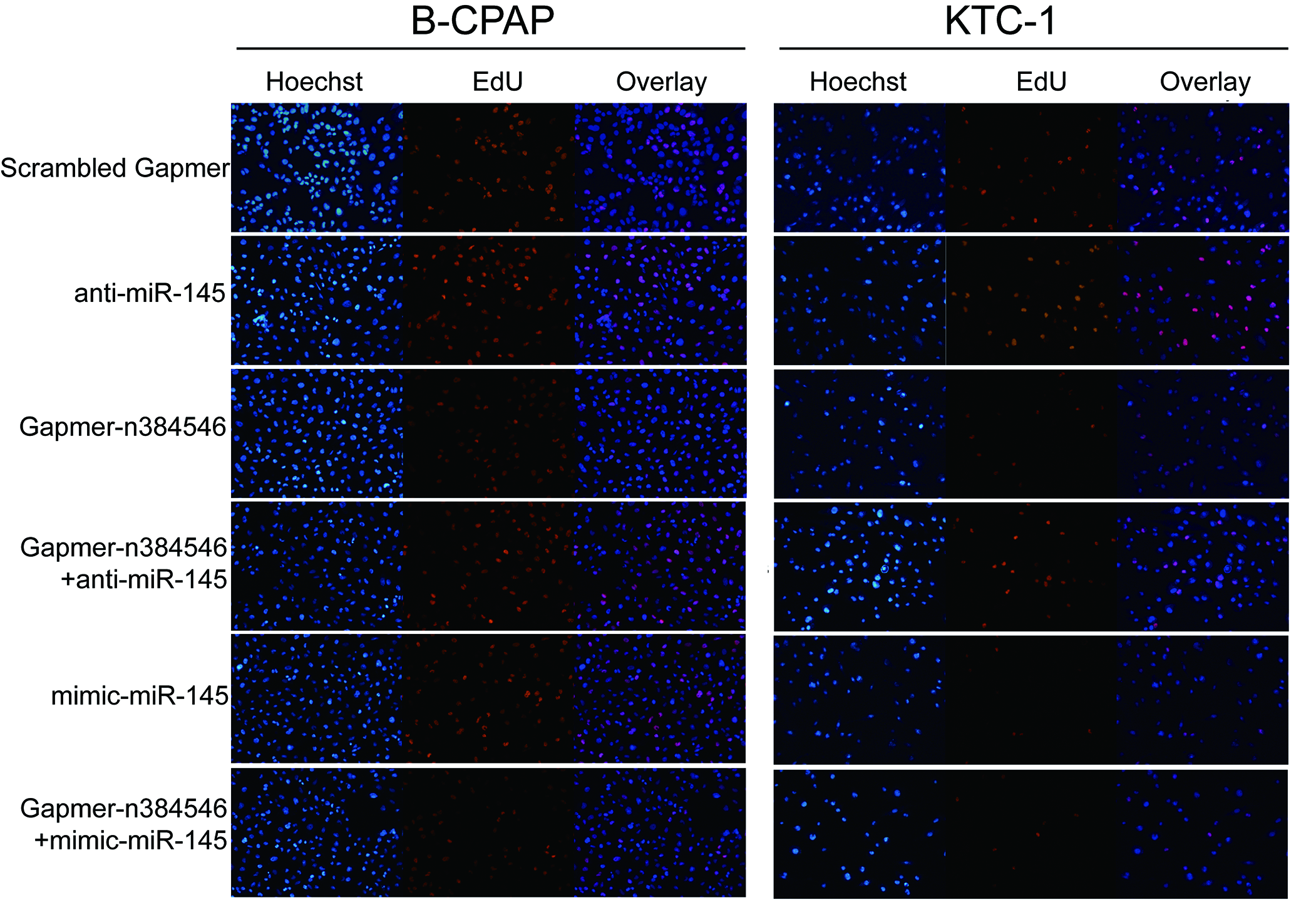

Supplement: Supplementary file 7 — Supplementary Figure 3 [file 41419_2019_1637_MOESM7_ESM.tif]

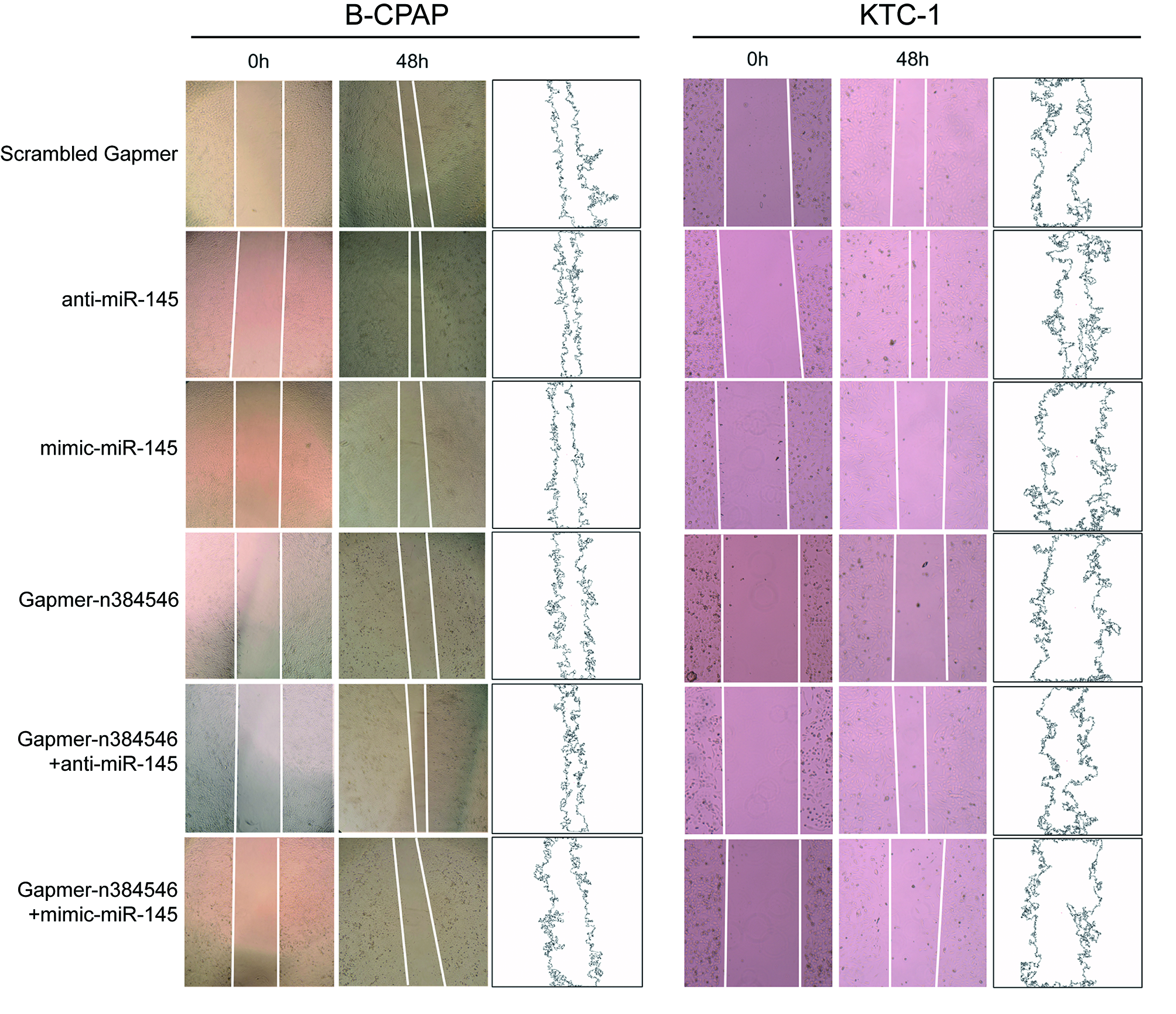

Supplement: Supplementary file 8 — Supplementary Figure 4 [file 41419_2019_1637_MOESM8_ESM.tif]

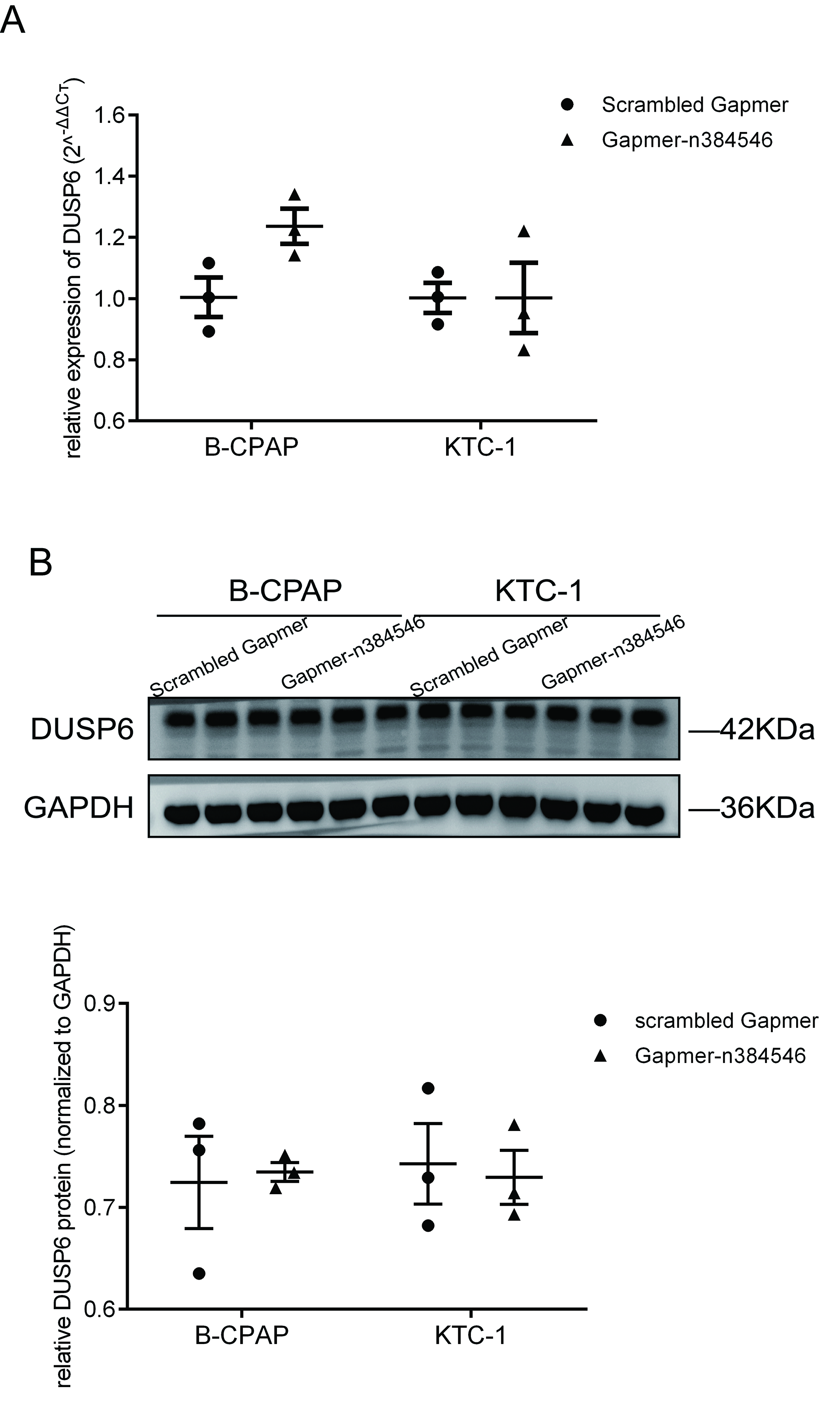

Supplement: Supplementary file 9 — Supplementary Figure 5 [file 41419_2019_1637_MOESM9_ESM.tif]
